# Supplementary material for: The Gene Curation Coalition: A global effort to harmonize gene-disease evidence resources
Source: Genet Med. Author manuscript; Available in PMC 2022 Aug 5. (PMC7613247; doi:10.1016/j.gim.2022.04.017)
Supplement: Supplementary materials [file EMS144755-supplement-Supplementary_materials.pdf]

Supplementary Figures:

Delphi Survey: Clinical Validity Term Refinement

	Evidence Terms	Likelihood Terms
After Survey 2 (N=38)	• Strong (33), High (3)<br>• Medium (-7), Moderate (26)<br>• Limited (23), Low (10), Minimal (-7)<br>• Ambiguous Evidence (-2), Disputed Evidence (19), Refuted Evidence (-3)<br>• No Evidence (-19), Refuted Evidence (29)<br>• Animal Model Only (27), No human evidence (12)<br>• No Claim (-21), No Disease Claim (-14), No Evidence (6), No Known Disease Link (11), No Known Disease Relationship (18), No Organismal evidence (-12), Undemonstrated (-19), Undetermined (-17)	• Confirmed (22), Definitive (14), Established (12), Validated (2), Verified (-7)<br>• Feasible (-24), Likely (5), Possible (-4), Potential (-4), Probable (12), Promising (-19), Provisional (-6)<br>• Implausible (-23), Improbable (-21), Insufficient (19), Low Confidence (-2), Possible (-12), Provisional (-19), Unconvincing (-21), Unlikely (-13)<br>• Challenged (-3), Contentious (-17), Contradicted (5), Controverted (-18), Disputed (21), Refuted (-5), Unconvincing (-17), Unlikely (-19)<br>• Disproven (20), Negated (-1), Refuted (24), Repudiated (-8)
Survey 3 (N= 241)	• **Strong (353)**, High (118)<br>• Medium (-47), **Moderate (242)**<br>• **Limited (197)**, Low (25), Minimal (41)<br>• Ambiguous Evidence (86), **Disputed Evidence (215)**, Refuted Evidence (-72)<br>• No Evidence (-54), **Refuted Evidence (244)**<br>• **Animal model only (270)**, No Human Evidence (131)<br>• No Evidence (147), No Known Disease Link (165), **No Known Disease Relationship (216)**	• **Confirmed (234)**, Definitive (216), Established (179), Validated (145)<br>• **Likely (139)**, Possible (20), Potential (-1), Probable (104), Provisional (-8)<br>• **Insufficient (177)**, Low Confidence (52), Possible (-82), Unlikely (-47)<br>• Challenged (8), Contradicted (138), **Disputed (201)**, Refuted (-69)<br>• Disproven (150), Negated (-48), **Refuted (185)**

Figure S1: Clinical Validity Term Refinement in the Delphi Survey

Likert scale answers were converted to point values and summed (Strongly Agree= 2points, Agree = 1 point, Neutral = 0 points, Disagree = -1 points, Strongly Disagree = -2 points). Each bullet point corresponds to a different clinical validity bucket. In Survey 2, terms with scores >2 standard deviations below the average score for each category were eliminated (denoted by red text) unless there were only two term options. In survey 3, the terms with the highest scores (denoted in bold) were chosen as the final term sets.

Finalized Term	Definition
Definitive[a]	The role of this gene in this particular disease has been repeatedly demonstrated in both the research and clinical diagnostic settings, and has been upheld over time (at least 2 independent publications over 3 years' time). No convincing evidence has emerged that contradicts the role of the gene in the specified disease.
Strong (Confirmed)	The role of this gene as a monogenic cause of disease has been repeatedly and independently demonstrated, providing very convincing evidence in humans and no conflicting evidence for this gene's role in this disease.
Moderate (Likely)	There is an intermediate amount of evidence in humans to support a causal role for this gene in this disease with no contradictory evidence. The body of evidence is not large (e.g. possibly only one key paper) but appears convincing enough that the gene-disease pair is likely to be validated with additional evidence in the near future.
Limited (Insufficient)	Little human evidence exists to support a causal role for this gene in this disease, but not all evidence has been refuted. For example, there may be a collection of rare missense variants in humans but without convincing functional impact, segregation data that could either arise by chance (e.g. across one or two meioses) or does not implicate a single gene, or functional data without direct recapitulation of the phenotype. Overall, the body of evidence does not meet contemporary criteria for claiming a valid association with disease. The majority are probably false associations.
Disputed Evidence	Although evidence has been reported, other evidence of equal weight challenges the claim.
Refuted Evidence	There has been an assertion of a gene-disease relationship in the literature, but new valid evidence has arisen that overturns the entire original body of evidence.
No known disease relationship	No disease claim in any organism has ever been made.
Animal model only	No (or very little) human disease evidence exists, but a convincing animal model exists.

Table S1. Definitions for gene curation categories. Harmonized definitions for gene-disease validity levels were drafted. They are listed here alongside the finalized chosen clinical validity term for each. [a]Definitive was not surveyed as a separate term choice

ClinGen	G2P	Orphanet	OMIM	PanelApp
Definitive	Confirmed	Present	Yes	Green
Strong	Confirmed	Present	Yes	Green
Moderate	Probable	Absent	Yes	Amber
Limited	Possible	Candidate	?Disease	Red
No Evidence	Absent	Absent	No Disease Claim	Red
Disputed	Absent	Candidate	?Disease	Red/Amber
Refuted	Absent	Absent (suppressed)	Reclassified-VUS	Red

Table S2. Clinical validity terms used by GenCC member groups before term harmonization

Supplementary Document: Delphi Survey Questions. The questions of the three rounds of the modified Delphi survey are provided below.

GenCC Survey Round 1

The GenCC Consortium is working to harmonize efforts around building and maintaining gene-level resources that define the role of genes in human disease. To best serve the community, we are seeking input on a harmonized set of terms and their definitions to then form a framework for standardization and collaboration. Please answer the following questions to help guide the use of terminology for defining the validity of a gene's role in disease.

Which resource do you represent? *

- ○ ClinGen
- ○ DDD/G2P
- ○ Orphanet
- ○ OMIM
- ○ PanelApp
- ○ TGMI
- ○ Other...

GenCC has discussed these survey terms in 2 in-person meetings and 4 web meetings. Approximately, how many of these have you attended? *

- None
- 25%
- 50%
- 100%
- Web meetings only
- In person meetings only

Which of the following terms do you feel best describes a gene that has unequivocally been implicated in disease (e.g. BRCA1 for breast cancer, CFTR for cystic fibrosis, etc)? Some terms relate to strength of evidence (e.g. strong, high) and others relate to the confidence of the claim (e.g. definitive). Please vote on the descriptor (e.g. definitive/strong) and we will later solicit input on the stem (e.g. disease gene, evidence, etc). *

- Strong (Evidence)
- High (Evidence)
- Confirmed (Disease Gene)
- Definitive (Disease Gene)
- Other...

Which of the following terms do you feel best describes a gene where there is a medium amount evidence in humans to support a causal role for this gene in a disease with no contradictory evidence. The body of evidence is not large (e.g. possibly only one key paper) but appears convincing enough that the gene-disease pair is likely to be validated with additional evidence in the near future. *

- Moderate (Evidence)
- Probable (Disease Gene)
- Likely (Disease Gene)
- Other...

Which of the following terms do you feel best describes a gene where little human evidence exists to support a causal role for this gene in this disease, but not all evidence has been refuted? For example, there may be a collection of rare missense variants in humans but without convincing functional impact, segregation data that could either arise by chance (e.g. across one or two meioses) or does not implicate a single gene, or functional data without direct recapitulation of the phenotype. Overall, the body of evidence does not meet contemporary criteria for claiming a valid association with disease. *

- Limited (Evidence)
- Minimal (Evidence)
- Insufficient (Evidence)
- Low Confidence (Disease Gene)
- Unlikely (Disease Gene)
- Other...

Do you feel it's important to distinguish between gene curation categories with insufficient evidence? For example, "Insufficient/Limited" versus "Refuted" which can be used to distinguish those genes that are at their earliest stage of evidence generation versus those for which no valid evidence has been presented despite a published claim. *

- ◯ Yes, require these categories to be distinguished
- ◯ Yes, but make these differences optional for groups performing curation
- ◯ No
- ◯ Unsure

Regardless of what you answered for the previous question, which of the following terms do you feel best describes a gene that although evidence has been reported, other evidence of equal weight challenges the claim? *

- ◯ Disputed (Evidence)
- ◯ Unlikely (Disease gene)
- ◯ Other...

Which of the following terms do you feel best describes a gene where no disease claim in any organism has ever been made? *

- No evidence
- No claim (Disease gene)
- Other...

Do you feel it’s important to distinguish between the two gene curation categories above? (i.e. if a gene has no human evidence, but a convincing animal model of the disease vs no evidence in any organism) *

- Yes
- No
- Unsure

Which of the following terms do you feel best describes a gene where there has been an assertion of a gene-disease association in the literature, but new valid evidence has arisen that overturns the original body of evidence? *

- Refuted (evidence)
- No evidence
- Other...

GenCC Survey Round 2

The GenCC Consortium is working to harmonize efforts around building and maintaining gene-level resources that define the role of genes in human disease. To best serve the community, we are seeking input on a harmonized set of terms and their definitions to then form a framework for standardization and collaboration. These terms may be used as a set to map across all represented curation efforts in the consortium. Please answer the following questions to help guide the use of terminology for defining the validity of a gene's role in disease.

Please check all that apply to you:

- [ ] Clinician (pediatrics, neurologist, etc.)
- [ ] Clinical Geneticist
- [ ] Genetic counselor
- [ ] Clinical lab
- [ ] Researcher

Where do you work? *

- ◯ United Kingdom
- ◯ United States
- ◯ Other...

Please answer about your type of work: *

	Research	Training	Administrati...	Clinical medi...	Counseling	Other
What is your ...	◯	◯	◯	◯	◯	◯
What is your ...	◯	◯	◯	◯	◯	◯

Are you a student? *

- ◯ Yes
- ◯ No

Where do you work? *

- ◯ Academia
- ◯ Industry
- ◯ Government
- ◯ Private practice
- ◯ Not-for-profit org.
- ◯ Other...

In what type of laboratory setting do you work? *

- ◯ Private diagnostic
- ◯ Private research
- ◯ University diagnostic
- ◯ University research
- ◯ Government
- ◯ NA

Are you a part of a consortia? If so, please choose or write in which one(s): *

- [ ] ClinGen
- [ ] DDD/G2P
- [ ] Orphanet
- [ ] OMIM
- [ ] PanelApp
- [ ] TGMI
- [ ] I am not part of a consortia.
- [ ] Other...

Describe your degree of acceptance of the following terms to describe a gene that has unequivocally been implicated in disease (e.g., BRCA1 for breast cancer, CFTR for cystic fibrosis, etc)? Some terms relate to strength of evidence (e.g., strong, high) and others relate to the confidence of the claim (e.g., definitive). Please vote on the descriptor (e.g., definitive/strong) and we will later solicit input on the stem (e.g., disease gene, evidence, etc). *

	Strongly disagree	Disagree	Agree	Strongly agree
Strong	☐	☐	☐	☐
High	☐	☐	☐	☐
Confirmed	☐	☐	☐	☐
Definitive	☐	☐	☐	☐
I would prefer anot...	☐	☐	☐	☐

If applicable, list your other preferred term

Short answer text

Describe your degree of acceptance of the following terms to describe a gene where there is a medium amount of evidence in humans to support a causal role for this gene in a disease with no contradictory evidence. The body of evidence is not large (e.g., possibly only one key paper with several families/probands and some functional data) but appears convincing enough that the gene-disease pair is likely to be validated with additional evidence in the near future. *

	Strongly disagree	Disagree	Agree	Strongly Agree
Moderate	☐	☐	☐	☐
Medium	☐	☐	☐	☐
Probable	☐	☐	☐	☐
Likely	☐	☐	☐	☐
Feasible	☐	☐	☐	☐
I would prefer anot...	☐	☐	☐	☐

If applicable, list your other preferred term

Short answer text

Describe your degree of acceptance of the following terms to describe a gene where little human evidence exists to support a causal role for this gene in this disease? For example, there may be rare missense variants in humans but without convincing functional impact, segregation data that could either arise by chance (e.g., across one or two meioses) or does not implicate a single gene, or functional data without direct recapitulation of the phenotype. Overall, the body of evidence does not meet contemporary criteria for claiming a valid association with disease. *

	Strongly disagree	Disagree	Agree	Strongly Agree
Limited	☐	☐	☐	☐
Low	☐	☐	☐	☐
Minimal	☐	☐	☐	☐
Insufficient	☐	☐	☐	☐
Low Confidence	☐	☐	☐	☐
Unlikely	☐	☐	☐	☐
Implausible	☐	☐	☐	☐
I would prefer anot...	☐	☐	☐	☐

If applicable, list your other preferred term

Short answer text

Do you feel it's important to distinguish between gene curation categories with insufficient evidence? For example, "Insufficient/Limited" versus "Refuted" which can be used to distinguish those genes that are at their earliest stage of evidence generation versus those for which evidence contradicts and outweighs the original report of a published claim . *

- Yes, require these categories to be distinguished
- Yes, but make these differences optional for groups performing curation
- No
- Unsure

Regardless of what you answered for the previous question, describe your degree of acceptance of the following terms to describe a gene that although evidence has been reported, other evidence of equal weight challenges the claim? *

	Strongly Disagree	Disagree	Agree	Strongly Agree
Disputed	☐	☐	☐	☐
Unlikely	☐	☐	☐	☐
Controverted	☐	☐	☐	☐
I would prefer anot...	☐	☐	☐	☐

If applicable, list your other preferred term

Short answer text

Describe your degree of acceptance of the following terms to describe a gene with a reported gene-disease association, but new valid evidence has arisen that overturns the original body of evidence? *

	Strongly Disagree	Disagree	Agree	Strongly Agree
Refuted	☐	☐	☐	☐
Repudiated	☐	☐	☐	☐
No evidence	☐	☐	☐	☐
I would prefer anot...	☐	☐	☐	☐

If applicable, list your preferred term

Short answer text

Describe your degree of acceptance of the following terms to describe a gene where no (or very little) human disease evidence exists (no human cases reported), but a convincing animal modeling the disease exists? *

	Strongly Disagree	Disagree	Agree	Strongly Agree
Animal model only	☐	☐	☐	☐
No human evidence	☐	☐	☐	☐
I would prefer anot...	☐	☐	☐	☐

If applicable, list your preferred term

Short answer text

Describe your degree of acceptance of the following terms to describe a gene where no disease claim in any organism has ever been made? *

	Strongly Disagree	Disagree	Agree	Strongly Agree
No evidence	☐	☐	☐	☐
No claim	☐	☐	☐	☐
No disease claim	☐	☐	☐	☐
Undemonstrated	☐	☐	☐	☐
I would prefer anot...	☐	☐	☐	☐

If applicable, list your preferred term

Short answer text

Do you feel it's important to distinguish between the two gene curation categories above? (i.e., if a gene has no human case observations, but a convincing animal model of the disease vs no evidence in any organism) *

- ◯ Yes
- ◯ No
- ◯ Unsure

In general, do you prefer terms that characterize the level of evidence supporting a claim (e.g strong, moderate, limited) or the likelihood that a gene is disease associated (eg. possible, likely, unlikely, etc)? *

- ◯ Evidence level
- ◯ Likelihood of association

Do you have any general comments?

Long answer text

GenCC Survey Round 3

Several groups and resources provide information that pertains to the validity of gene-disease relationships; however, the standards and terminologies to define the evidence base for a gene's role in disease are still evolving. To tackle this issue, the Gene Curation Coalition (GenCC) was formed including members of the Clinical Genome Resource (ClinGen), Deciphering Developmental Disorders/Gene2Phenotype (DDD/G2P), Genetics Home Reference (GHR), Genomics England PanelApp (PanelApp), Online Mendelian Inheritance in Man (OMIM), Orphanet, and Transforming Genetic Medicine Initiative (TGMI). Together, this group is working to harmonize approaches to ensure gene-level resources are comparable and interoperable. This allows groups to most effectively work together and provide consistent and useful resources for the community.

The most common use case for terms generated by this survey is to determine which genes should go on a gene panel in a variety of different contexts. This includes clinical diagnostic testing for monogenic disease as well as presymptomatic testing and other healthy screening. For the purposes of this survey, all terms being considered are in the context of genes implicated in highly penetrant, monogenic disease.

To best serve the community, we are seeking input on the harmonized set of terms and their definitions, to then form a framework for standardization and collaboration. These terms will be used as the recommended set and to map all other terms used across the represented curation efforts in the consortium. Please answer the following questions to help guide the development of the terminology for defining the validity of a gene's role in monogenic disease. This survey should take 10-15 minutes to complete.

NOTE: We suggest that survey respondents watch a 6 minute optional explanatory video that discusses the purpose of this work before accessing and completing the survey. The video can be found here: https://vimeo.com/306463165

Terms Survey

Please check all that apply to you: *

- [ ] Physician
- [ ] Medical Genetics Physician
- [ ] Genetic Counselor
- [ ] Clinical Genetics Laboratory Director or Staff
- [ ] Researcher
- [ ] Scientific Curator
- [ ] Other: ____________________

In what country do you work? *

- ○ Australia
- ○ Canada
- ○ France
- ○ United Kingdom
- ○ United States
- ○ Other: ____________________

Please answer about your type of work. What is your primary type of work? *

- ◯ Research
- ◯ Training
- ◯ Administration
- ◯ Clinical medicine
- ◯ Counseling
- ◯ Database curation
- ◯ Other: ______

What is your secondary type of work? *

- ◯ Research
- ◯ Training
- ◯ Administration
- ◯ Clinical medicine
- ◯ Counseling
- ◯ Database curation
- ◯ Other: ______

Where do you work? *

- ◯ Academia
- ◯ Industry
- ◯ Government
- ◯ Private practice
- ◯ Not-for-profit organization
- ◯ Other: ____________

In what type of laboratory setting do you work? *

- ◯ Private diagnostic
- ◯ Private research
- ◯ University diagnostic
- ◯ University research
- ◯ Government
- ◯ N/A

Are you working within one or more of the following programs? If so, please choose or write in which one(s):

- [ ] ClinGen
- [ ] DDD/G2P
- [ ] GA4GH
- [ ] OMIM
- [ ] Orphanet
- [ ] PanelApp
- [ ] TGMI
- [ ] Not in a consortium
- [ ] Other: ____________

On a scale of 1 to 5, what is your familiarity with human genetics? *

	1	2	3	4	5	
Unfamiliar	○	○	○	○	○	Very familiar. I work in the field or am closely tied.

In examining approaches to defining gene-disease validity, some terms address the strength of evidence supporting the claim (e.g. strong, moderate, limited) and others address the confidence in, or likelihood of, a gene having a valid relationship with disease (definitive, confirmed, probable, possible, unlikely, disputed, refuted, etc). Please describe your degree of acceptance for each approach. *

	Strongly Disagree	Disagree	Neutral	Agree	Strongly Agree
Strength of evidence	◯	◯	◯	◯	◯
Confidence/Likelihood	◯	◯	◯	◯	◯

Please describe your degree of acceptance for choosing one of these approaches versus supporting both systems which could be used jointly or in different contexts: *

	Strongly Disagree	Disagree	Neutral	Agree	Strongly Agree
Support both systems	◯	◯	◯	◯	◯
Choose one system	◯	◯	◯	◯	◯

Please note, for all questions that follow, all terms would be used in the context of a specific disease relationship and a given gene may have different levels of evidence for different diseases. Also, these terms relate to the strength of the evidence/likelihood of disease association and do not address the penetrance or expressivity or clinical impact of the disease or phenotype.

Regardless of which terminology system(s) you prefer, please answer the questions regarding terms that characterize the level of evidence or likelihood of a gene disease relationship:

Describe your degree of acceptance of the following terms to describe a gene that has unequivocally been implicated in disease (e.g., BRCA1 for breast cancer, CFTR for cystic fibrosis, etc)? *

Level of evidence terms:

	Strongly Disagree	Disagree	Neutral	Agree	Strongly Agree
Strong	○	○	○	○	○
High	○	○	○	○	○

*

Likelihood terms:

	Strongly Disagree	Disagree	Neutral	Agree	Strongly Agree
Confirmed	○	○	○	○	○
Definitive	○	○	○	○	○
Established	○	○	○	○	○
Validated	○	○	○	○	○

ClinGen distinguishes between genes that have a strong level of evidence but may be newly discovered (classified as Strong) and those that have stood the test of time (at least 3 years), and have been replicated in multiple studies to ensure no refuting evidence arises (classified as Definitive). Regardless of the terms used, do you think this distinction is useful? *

- ○ Strongly Disagree
- ○ Disagree
- ○ Neutral
- ○ Agree
- ○ Strongly Agree

Describe your degree of acceptance of the following terms to describe a gene where there is an intermediate amount of evidence in humans to support a causal role for this gene in a disease with no contradictory evidence. The body of evidence is not large (e.g., possibly only one key paper with several families/probands and some functional data) but appears convincing enough that the gene-disease pair is likely to be validated with additional evidence in the near future. *

Level of evidence terms:

	Strongly Disagree	Disagree	Neutral	Agree	Strongly Agree
Medium	◯	◯	◯	◯	◯
Moderate	◯	◯	◯	◯	◯

*

Likelihood terms:

	Strongly Disagree	Disagree	Neutral	Agree	Strongly Agree
Likely	◯	◯	◯	◯	◯
Possible	◯	◯	◯	◯	◯
Potential	◯	◯	◯	◯	◯
Probable	◯	◯	◯	◯	◯
Provisional	◯	◯	◯	◯	◯

Describe your degree of acceptance of the following terms to describe a gene where little human evidence exists to support a causal role for the gene in a given disease? For example, there may be rare missense variants in humans but without convincing functional impact, segregation data that could either arise by chance (e.g., across one or two meioses) or does not implicate a single gene, or functional data without direct recapitulation of the phenotype. Overall, the body of evidence does not meet contemporary criteria for claiming a valid relationship with disease. *

Level of evidence terms:

	Strongly Disagree	Disagree	Neutral	Agree	Strongly Agree
Limited	○	○	○	○	○
Low	○	○	○	○	○
Minimal	○	○	○	○	○

*

Likelihood terms:

	Strongly Disagree	Disagree	Neutral	Agree	Strongly Agree
Insufficient	○	○	○	○	○
Low Confidence	○	○	○	○	○
Possible	○	○	○	○	○
Unlikely	○	○	○	○	○

Do you feel it's important to distinguish between gene curation categories with insufficient evidence? For example, "Insufficient/Limited" versus "Disputed/Refuted" which can be used to distinguish those genes that are at their earliest stage of evidence generation versus those for which evidence contradicts and may outweigh the original report of a published claim. *

- ○ Yes, require these categories to be distinguished
- ○ Yes, but make these differences optional for groups performing curation
- ○ No
- ○ Unsure

Regardless of how you answered the previous question, describe your degree of acceptance of the following terms to describe a gene that, although evidence has been reported, other evidence of equal weight challenges the claim, or the full body of evidence does not meet a level to ever have proposed a causal relationship with disease?

Level of evidence terms:

	Strongly Disagree	Disagree	Neutral	Agree	Strongly Agree
Ambiguous evidence	◯	◯	◯	◯	◯
Disputed evidence	◯	◯	◯	◯	◯
Refuted evidence	◯	◯	◯	◯	◯

*

Likelihood terms:

	Strongly Disagree	Disagree	Neutral	Agree	Strongly Agree
Challenged	◯	◯	◯	◯	◯
Contradicted	◯	◯	◯	◯	◯
Disputed	◯	◯	◯	◯	◯
Refuted	◯	◯	◯	◯	◯

Describe your degree of acceptance of the following terms to describe a gene with a reported gene-disease relationship, but new valid evidence has arisen that overturns the original body of evidence (e.g. all variants implicated in disease have now been found at high frequency in other populations; different genes now explain disease in all reported families)? *

Level of evidence terms:

	Strongly Disagree	Disagree	Neutral	Agree	Strongly Agree
No evidence	○	○	○	○	○
Refuted evidence	○	○	○	○	○

*

Likelihood terms:

	Strongly Disagree	Disagree	Neutral	Agree	Strongly Agree
Disproven	○	○	○	○	○
Negated	○	○	○	○	○
Refuted	○	○	○	○	○

Describe your degree of acceptance of the following terms to describe a gene where no human disease evidence exists (no human cases reported), but a convincing animal model of the disease exists? *

	Strongly Disagree	Disagree	Neutral	Agree	Strongly Agree
Animal model only	○	○	○	○	○
No human evidence	○	○	○	○	○
Other: please write in a term below	○	○	○	○	○

If applicable, write in your "other" preferred term:

Your answer

Describe your degree of acceptance of the following terms to describe a gene where no disease claim in any organism has ever been made? *

	Strongly Disagree	Disagree	Neutral	Agree	Strongly Agree
No evidence	○	○	○	○	○
No known disease link	○	○	○	○	○
No known disease relationship	○	○	○	○	○

Do you feel it's important to distinguish between the two gene curation categories above? (i.e., if a gene has no human case observations, but a convincing animal model of the disease vs no evidence in any organism) *

- ○ Yes
- ○ No
- ○ Unsure

Which of these categories of genes do you feel should go on diagnostic testing panels (that is, panels used to genetically diagnose affected individuals)? *

- [ ] Genes that has unequivocally been implicated in disease (e.g., BRCA1 for breast cancer, CFTR for cystic fibrosis, etc)
- [ ] Genes where there is an intermediate amount of evidence in humans to support a causal role for this gene in a disease with no contradictory evidence. The body of evidence is not large (e.g., possibly only one key paper with several families/probands and some functional data) but appears convincing enough that the gene-disease pair is likely to be validated with additional evidence in the near future.
- [ ] Genes where little human evidence exists to support a causal role for the gene in a given disease. For example, there may be rare missense variants in humans but without convincing functional impact, segregation data that could either arise by chance (e.g., across one or two meioses) or does not implicate a single gene, or functional data without direct recapitulation of the phenotype. Overall, the body of evidence does not meet contemporary criteria for claiming a valid relationship with disease.
- [ ] Genes that, although evidence has been reported, other evidence of equal weight challenges the claim, or the full body of evidence does not meet a level to ever have proposed a causal relationship with disease.
- [ ] Genes with a reported gene-disease relationship, but new valid evidence has arisen that overturns the original body of evidence (e.g. all variants implicated in disease have now been found at high frequency in other populations; different genes now explain disease in all reported families).
- [ ] No opinion
- [ ] Not informed enough to make a response

Which of these categories of genes do you feel should be included in tests offered to healthy individuals (e.g., carrier screening, preventive risk assessment)? *

- [ ] Genes that has unequivocally been implicated in disease (e.g., BRCA1 for breast cancer, CFTR for cystic fibrosis, etc)
- [ ] Genes where there is an intermediate amount of evidence in humans to support a causal role for this gene in a disease with no contradictory evidence. The body of evidence is not large (e.g., possibly only one key paper with several families/probands and some functional data) but appears convincing enough that the gene-disease pair is likely to be validated with additional evidence in the near future.
- [ ] Genes where little human evidence exists to support a causal role for the gene in a given disease. For example, there may be rare missense variants in humans but without convincing functional impact, segregation data that could either arise by chance (e.g., across one or two meioses) or does not implicate a single gene, or functional data without direct recapitulation of the phenotype. Overall, the body of evidence does not meet contemporary criteria for claiming a valid relationship with disease.
- [ ] Genes that, although evidence has been reported, other evidence of equal weight challenges the claim, or the full body of evidence does not meet a level to ever have proposed a causal relationship with disease.
- [ ] Genes with a reported gene-disease relationship, but new valid evidence has arisen that overturns the original body of evidence (e.g. all variants implicated in disease have now been found at high frequency in other populations; different genes now explain disease in all reported families).
- [ ] No opinion
- [ ] Not informed enough to make a response

Which of these categories of genes do you feel should go on exome/genome sequencing of individuals with suspected monogenic disease? *

- [ ] Genes that has unequivocally been implicated in disease (e.g., BRCA1 for breast cancer, CFTR for cystic fibrosis, etc)
- [ ] Genes where there is an intermediate amount of evidence in humans to support a causal role for this gene in a disease with no contradictory evidence. The body of evidence is not large (e.g., possibly only one key paper with several families/probands and some functional data) but appears convincing enough that the gene-disease pair is likely to be validated with additional evidence in the near future.
- [ ] Genes where little human evidence exists to support a causal role for the gene in a given disease. For example, there may be rare missense variants in humans but without convincing functional impact, segregation data that could either arise by chance (e.g., across one or two meioses) or does not implicate a single gene, or functional data without direct recapitulation of the phenotype. Overall, the body of evidence does not meet contemporary criteria for claiming a valid relationship with disease.
- [ ] Genes that, although evidence has been reported, other evidence of equal weight challenges the claim, or the full body of evidence does not meet a level to ever have proposed a causal relationship with disease.
- [ ] Genes with a reported gene-disease relationship, but new valid evidence has arisen that overturns the original body of evidence (e.g. all variants implicated in disease have now been found at high frequency in other populations; different genes now explain disease in all reported families).
- [ ] No opinion
- [ ] Not informed enough to make a response

Which of these categories of genes do you feel should go on exome/genome sequencing of individuals with suspected monogenic disease? *

- [ ] Genes that has unequivocally been implicated in disease (e.g., BRCA1 for breast cancer, CFTR for cystic fibrosis, etc)
- [ ] Genes where there is an intermediate amount of evidence in humans to support a causal role for this gene in a disease with no contradictory evidence. The body of evidence is not large (e.g., possibly only one key paper with several families/probands and some functional data) but appears convincing enough that the gene-disease pair is likely to be validated with additional evidence in the near future.
- [ ] Genes where little human evidence exists to support a causal role for the gene in a given disease. For example, there may be rare missense variants in humans but without convincing functional impact, segregation data that could either arise by chance (e.g., across one or two meioses) or does not implicate a single gene, or functional data without direct recapitulation of the phenotype. Overall, the body of evidence does not meet contemporary criteria for claiming a valid relationship with disease.
- [ ] Genes that, although evidence has been reported, other evidence of equal weight challenges the claim, or the full body of evidence does not meet a level to ever have proposed a causal relationship with disease.
- [ ] Genes with a reported gene-disease relationship, but new valid evidence has arisen that overturns the original body of evidence (e.g. all variants implicated in disease have now been found at high frequency in other populations; different genes now explain disease in all reported families).
- [ ] No opinion
- [ ] Not informed enough to make a response

Please rank the term sets: *

	First Choice	Second Choice	Third Choice	Fourth Choice	Fifth Choice
Definitive/Stron...	○	○	○	○	○
Strong/Modera...	○	○	○	○	○
Strong/Modera...	○	○	○	○	○
Confirmed/Like...	○	○	○	○	○
Confirmed/Pro...	○	○	○	○	○

If none of these options represent your top pick "set", please write out your own set below, choosing from all options listed above

Short answer text

Do you have any general comments about our harmonization efforts?

Long answer text

If you are willing to be contacted for additional feedback, please include your name and email.

Short answer text
